# Genomic Characterization and gE/gI-Deleted Strain Construction of Novel PRV Variants Isolated in Central China

**DOI:** 10.3390/v15061237

**Published:** 2023-05-25

**Authors:** Jianle Ren, Shanshan Tan, Xinxin Chen, Jiying Yao, Zhihong Niu, Ying Wang, Lei Ma, Xiaolong Gao, Sheng Niu, Libin Liang, Junping Li, Yujun Zhao, Wen-xia Tian

**Affiliations:** 1 College of Veterinary Medicine, Shanxi Agricultural University, Jinzhong 030801, China; renjianle0615@163.com (J.R.); tanshanshan2021@163.com (S.T.); yjy2323126320@163.com (J.Y.); nzh1217899264@163.com (Z.N.); wangying@sxau.edu.cn (Y.W.); niusheng@sxau.edu.cn (S.N.); lianglibin521@126.com (L.L.); lijunping916@163.com (J.L.); tgzhaoyujun@163.com (Y.Z.); 2 Beijing Solarbio Science & Technology Co., Ltd., Beijing 101102, China; jjdwswx@126.com; 3 School of Biotechnology and Food Engineering, Anyang Institute of Technology, Anyang 455000, China; nihaowsml2008@163.com; 4 Beijing Animal Disease Prevention and Control Center, Beijing 102629, China; veterinary89@126.com

**Keywords:** PRV variant, variation, vaccine, Bartha-K61, immunity protection

## Abstract

Pseudorabies virus (PRV) variants have caused substantial economic losses in the swine industry in China since 2011. To surveil the genetic variation in PRV field strains, here, two novel variant strains of PRV were isolated from Shanxi Province in central China and were designated SX1910 and SX1911. To identify the genetic characteristics of the two isolates, their complete genomes were sequenced, and phylogenetic analysis and sequence alignment revealed that field PRV variants have undergone genetic variations; notably, the protein-coding sequences UL5, UL36, US1 and IE180 exhibited extensive variation and contained one or more hypervariable regions. Furthermore, we also found that the glycoproteins gB and gD of the two isolates had some novel amino acid (aa) mutations. Importantly, most of these mutations were located on the surface of the protein molecule, according to protein structure model analysis. We constructed a mutant virus of SX1911 with deletion of the gE and gI genes via CRISPR/Cas9. When tested in mice, SX1911-ΔgE/gI-vaccinated mice were protected within a comparable range to Bartha-K61-vaccinated mice. Additionally, a higher dose of inactivated Bartha-K61 protected the mice from lethal SX1911 challenge, while a lower neutralization titer, higher viral load and more severe microscopic lesions were displayed in Bartha-K61-vaccinated mice. These findings highlight the need for continuous monitoring of PRV and novel vaccine development or vaccination program design for PRV control in China.

## 1. Introduction

Pseudorabies virus (PRV) is a double-stranded DNA virus that belongs to the family Herpesviridae, subfamily Alphaherpesvirinae, and genus Varicellovirus [1,2,3]. This virus is the etiological agent of pseudorabies (PR) or Aujeszky’s disease [4]. PRV exhibits a broad host range and is capable of infecting most mammals and some avian species, but pigs are the natural host of the virus [5,6,7]. PRV infections cause neurological and respiratory system disorders, causing high mortality in newborn piglets and reproductive disease in pregnant sows [5]. In particular, PRV can establish life-long latent infection in the neurological tissues and lymphoid tissues of recovered pigs, leading to recurrent infection [1,8,9].

Inactivated or attenuated gene-deletion vaccines, along with differential diagnosis, has proven to be the best strategy to control and eradicate PRV [6,7,10]. Due to the control efforts and strict implementation of this strategy, PRV has disappeared from domestic pig populations in several parts of the world, such as New Zealand, most European countries and the USA [6,11]. In China, the attenuated vaccine strain Bartha-K61 was imported the 1980s and has been broadly applied since, resulting in relatively favorable control of PR [5,12]. However, PR reemerged in large-scale swine herds in most regions of China in 2011 [13,14]. Subsequently, the causative agent was confirmed to be PRV variant strains. Compared with classical strains, variant strains exhibit increased virulence, causing high mortality in newborn piglets and finishing pigs and an increased abortion rate in sows [15,16,17]. Of note, the Bartha-K61 vaccine could not provide effective protection against PRV variants [18,19,20,21]. Genetically, PRV can be divided into two genotypes. PRV strains in China belong to genotype II, while genotype I comprises mainly strains isolated in America and Europe [22]. Within genotype II, PRV variant genomes also exhibit marked sequence divergence in comparison to classical strains [22,23,24]. In the last decade, PRV variants have undergone extensive intragenotype and intergenotype recombination among strains and amino acid (aa) mutation, such as that of viral glycoproteins or other viral proteins [25,26,27]. More PRV variants have also been isolated from cows, dogs, sheep, wolves, minks and even humans [28,29,30,31]. In this regard, recombination events and aa mutations might alter viral virulence or the immune response of novel hosts and lead to rapid interspecies transmission [26]. Thus, it is necessary to continuously monitor and analyze epidemic or genetic variation in PRV in the future, which will help facilitate PRV prevention and control.

In this study, we successfully isolated two novel PRV strains, SX1910 and SX1911, from the lung tissue samples of sick piglets on pig farms in Shanxi Province, China. By analyzing the genetic variation in and evolution of the isolates, we found that SX1910 and SX1911 were classified into genotype II and were variant strains. Furthermore, we revealed that the PRV variants isolated from the field were undergoing genetic variations, and the aa sequences of the viral proteins UL5, UL36, US1 and IE180 exhibited extensive variations, including one or more hypervariable regions. Of note, novel aa mutations in gB and gD were found in SX1910 and SX1911. Most of these mutations were located on the surface of the molecule, according to protein structure model analysis. Based on the PRV strain SX1911, we generated a mutant virus lacking the gE and gI genes (SX1911-ΔgE/gI) via CRISPR/Cas9 and the LoxP system platform. Immunization and challenge tests in mice indicated that inactivated SX1911-ΔgE/gI could provide comparable protection in comparison with inactivated Bartha-K61. However, the viral loads and microscopic lesions of Bartha-K61-vaccinated mice were much higher and more severe, respectively, than those of mice inoculated with SX1911-ΔgE/gI. These findings will help us understand the epidemic status of PRV variants and will have important implications for PRV control in China.

## 2. Materials and Methods

### 2.1. Cells, Viruses and Antibodies

Vero-CCL81 cells (ATCC, CCL81) were cultured in Dulbecco’s modified Eagle’s medium (DMEM) (Gibco, Grand Island, NY, USA) supplemented with 10% fetal bovine serum (FBS) (Gibco, USA) at 37 °C in a 5% CO_2_ incubator. The two PRV variant strains SX1910 (GenBank no. OL606749.1) and SX1911 (GenBank no. OP376823.1) were isolated from PRV-infected piglets from pig farms in Shanxi Province in 2019. The PRV Bartha-K61 vaccine strain (Genbank no. JF797217.1) was a gift from Shanxi LUCKIER Biopharmaceutical Co., Ltd. and was preserved in our laboratory. The PRV strains were propagated in Vero-CCL81 cells maintained in DMEM supplemented with 2% FBS (Gibco, USA) at 37 °C with 5% CO_2_. The anti-PRV gB monoclonal antibody was a gift from Dr. Jiangwei Song (Institute of Animal Husbandry and Veterinary Medicine, Beijing Academy of Agriculture and Forestry Sciences).

### 2.2. Virus Isolation and Identification

Lung tissues that were positive for PRV were homogenized in Dulbecco’s modified Eagle’s medium. The homogenates were centrifuged and filtered through a 0.22 µm filter to remove bacteria, and inoculated onto Vero cells. Then, the cells were incubated at 37 °C and examined daily for a cytopathic effect (CPE). After the CPE appeared, the infected cells were characterized via immunofluorescence assays (IFAs) using an anti-PRV gB protein monoclonal antibody and PCR targeting of the gE gene (Table S1). Finally, the viruses were purified via plaque purification, with homogeneity monitored based on the plaque sizes.

### 2.3. Multistep Growth Analysis

The multistep growth kinetics of PRV were measured as previously described [21]. Briefly, Vero cells were infected with PRV at a multiplicity of infection (MOI) of 0.01. After absorption for 1 h at 37 °C, the unbound viruses were removed via brief acid and PBS washing. The cells were washed twice with PBS. Then, the cells were supplemented with fresh DMEM containing 2% FBS. The cell cultures were collected at the indicated times post-infection, and the virus titers are expressed as the 50% tissue culture infectious dose (TCID_50_), as determined according to the Reed–Muench method [32].

### 2.4. Plaque Size Analysis

To analyze the size of the plaques induced by PRV, the virus supernatants were diluted with DMEM at a 10-fold dilution, transferred to 6-well plates containing Vero cell monolayers at a volume of 1 mL/well and incubated to allow absorption at 37 °C for 1 h. Then, the supernatants were removed by washing the cells twice with PBS, and the cells were overlaid with 2 mL of DMEM containing 0.5% methylcellulose. After incubation at 37 °C for 60 h, the plaque sizes were analyzed using ImageJ2X software (National Institutes of Health, Bethesda, MD, USA) after staining with 4% paraformaldehyde containing 0.1% crystal violet.

### 2.5. Genomic Sequencing

PRV genomic DNA was extracted from infected Vero cells as previously described [33]. Five micrograms of genomic DNA were submitted to Sangon Biotech (Shanghai, China) Co., Ltd. for next-generation sequencing (NGS) using an Illumina HiSeq^TM^. After the host sequences were removed, the raw assembled genomes were aligned using Blast software (NCBI) to analyze the spanned sequences. Then, the sequences corresponding to the remaining gaps were amplified via PCR, and the products were cloned into the pEASY-Blunt vector (TransGen, Beijing, China) for Sanger sequencing. Following assembly and annotation, the genome sequences of PRV strains SX1910 and SX1911 were submitted to the GenBank database under accession numbers OL606749.1 and OP376823.1, respectively.

### 2.6. Phylogenetic Analysis and Sequence Alignment

For PRV, the gC gene and whole genome have been most widely used for phylogenetic analysis. Thus, the gC gene sequences (Table S2) were collected from the GenBank database and input into MEGA 5.1 software (Mega Limited, Auckland, New Zealand) for analysis using the neighbor-joining algorithm, 1000 bootstrap replicates and the Kimura 2-parameter substitution model. Additionally, whole-genome phylogenetic analysis was performed using Geneious Prime software (version 2022.2, Biomatters, Auckland, New Zealand) with the neighbor-joining method, 100 bootstrap replicates and the Tamura–Nei genetic distance model.

The whole genomes of the SX1910 and SX1911 strains were aligned with those of eight reference PRV genomes, including the European–American strain Bartha-K61, the classical strain Fa, and variant strains (TJ, GD0304, HN1201, JS-2012 and HeN1), using the mVista genomic analysis tool (http://genome.lbl.gov/vista/mvista/submit.shtml, accessed on 29 October 2022) with global LAGAN alignment. Moreover, the selected gene-encoding sequences (gB, gC, gD, UL5, UL36, US1 and IE180) of the PRV strains were aligned via Geneious Prime software (version 2022.2, Biomatters, Auckland, New Zealand) with the Clustal Omega program. The reference strains used for alignment are listed in Table S2.

### 2.7. Construction of a gE/gI-Deleted Virus

sgRNAs targeting the gE and gI genes were designed using an online CRISPR tool (https://www.genscript.com/gRNA-design-tool.html, accessed on 15 May 2021). The sgRNA CRISPR/Cas9 plasmid was constructed as previously described [21]. In brief, the oligo pairs were synthesized (Table S3) and annealed under the following conditions: 5 min at 95 °C and 30 min at 25 °C. The purified product was then cloned into the plasmid pX335 (sgRNA/Cas9 expression vector) at the BbsI restriction site, followed by verification via DNA sequencing.

To delete gE and gI, the homologous arms were amplified from the variant strain SX1911. Two pairs of primers containing the Loxp site were designed (Table S3) and used to amplify GFP from the plasmid pEGFP-N2 (Clontech, Mountain View, CA, USA) via PCR using Phanta^®^ Max Super-Fidelity DNA Polymerase (Vazyme, Nanjing, China). A donor GFP construct flanked by homologous arms was then constructed via overlapping PCR. To generate the recombinant virus, PRV genomic DNA was extracted from infected Vero cells as previously described [34], and cotransfected with linear donor DNA (5.0 µg) and two sgRNA plasmids (1.5 µg each) into Vero cells using Lipofectamine 2000 Reagent (Invitrogen, Carlsbad, CA, USA). The CPE was monitored daily, and the recombinant virus carrying GFP was harvested 72 h later. To delete the GFP gene, the genomic DNA of the recombinant virus and plasmid expressing Cre were cotransfected into Vero cells. Then, gE/gI-deleted viruses were screened by determining the loss of GFP fluorescence. All viruses were purified via plaque purification, with homogeneity monitored using the plaque sizes, and were verified via DNA sequencing.

### 2.8. Experiments in Mice

#### 2.8.1. Pathogenicity Test

One hundred and thirty 6-week-old female SPF Kunming mice were randomly divided into 13 groups with ten mice in each group. Mice in groups 1–4, 5–8 and 9–12 were subcutaneously inoculated with 100 μL of different doses (10^3^, 10^4^, 10^5^ or 10^6^ TCID_50_) of SX1910, SX1911 and SX1911ΔgE/gI, respectively. Mice in group 13 were injected with DMEM as a mock control. Clinical signs were observed daily, and the number of deaths and time of death of mice in each group were recorded. At 14 days post-challenge (dpc), all surviving mice were euthanized. The 50% lethal dose (LD_50_) was calculated using the Reed–Muench method [32]. Additionally, a detailed description of the calculation of clinical scores is shown in Table 1.

#### 2.8.2. Immunization and Challenge Test

Fifty-four 6-week-old female SPF Kunming mice were randomly divided into 6 groups with nine mice in each group. The mice in groups 1–2 were inoculated with 200 μL of inactivated SX1911-ΔgE/gI at a dose of 10^6^ TCID_50_/mL or 10^7^ TCID_50_/mL via both the subcutaneous and intramuscular routes. Similarly, the mice in groups 3–4 were injected with Bartha-K61. Mice in the unvaccinated group (positive control) and negative control group received 200 μL of DMEM, respectively. Following immunization, the clinical symptoms were recorded daily. At 14 days post-immunization (dpi), mice in groups 1–4 received a second immunization. At 28 dpi, serum samples were collected to monitor PRV-neutralizing antibody production. Then, all mice were challenged subcutaneously (i.p.) with PRV SX1911 at a dose of 200 μL of 10^6^ TCID_50_, except for those in the negative control group. After the challenge, the clinical signs of disease were recorded and scored daily. At 14 dpc, all surviving mice were euthanized and necropsied, and the lungs were collected for viral load and histopathology analyses.

### 2.9. Quantitative PCR (qPCR)

The viral tissue load was measured via absolute quantitative PCR (qPCR) targeting the gB gene on a QuantStudio^TM^ 5 Real-Time PCR Instrument (Applied Biosystem, Carlsbad, CA, USA) using previously described primers and following the manufacturer’s recommendations [21]. According to the standard curve, the viral loads and virus shedding were calculated and expressed as log10 copies per mouse.

### 2.10. Histopathological Examinations

Histopathological examination was performed as previously described [21]. Briefly, the collected lungs were fixed with a 4% paraformaldehyde solution at room temperature for 48 h. The fixed tissues were dehydrated in graded alcohol and embedded in paraffin wax. Microsections were cut, stained with hematoxylin and eosin (HE) and examined via light microscopy to identify microscopic pathological changes.

### 2.11. Statistical Analyses

Statistical analyses were performed using a one-way or two-way analysis of variance (ANOVA) in GraphPad Prism 5 (San Diego, CA, USA). *p* values < 0.05 were considered to indicate statistical significance; *p* values < 0.001 were considered to indicate extreme significance.

## 3. Results

### 3.1. Isolation and Identification of Virus

**Figure 1:**
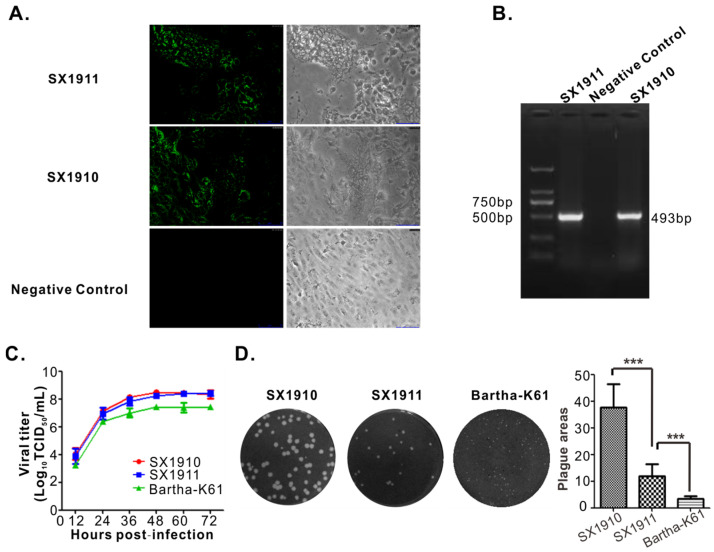
Identification of the isolated PRV strains. (**A**) Indirect immunofluorescence assay (IFA) for the detection of the PRV strains SX1910 and SX1911 (magnification 200X). Vero cells infected with the supernatant from cultures exhibiting a CPE were fixed at 48 h post-inoculation and examined via IFA using monoclonal antibodies against the gB protein. (**B**) PCR verification of the PRV strains SX1910 and SX1911. Vero cells were inoculated with the supernatant from cultures exhibiting a CPE and subjected to PCR amplification using the primer pair gE-F/gE-R specific for the PRV gE gene. (**C**) Multistep growth curve of SX1910 and SX1911 compared with Bartha-K61 in Vero cells. (**D**) Plaque sizes of SX1910 and SX1911 compared with Bartha-K61 in Vero cells. Data are presented as the mean ± SD, and an asterisk indicates a significant difference ***: *p* < 0.001.

PRV-positive lung tissue homogenates were centrifuged, filtered through a 0.22 µm filter to remove bacteria, and inoculated onto Vero cells. A distinct CPE was characterized by rounded cells at 36 h post-inoculation. When the Vero cells were infected with supernatant from cultures exhibiting a CPE, a positive signal for the PRV gB protein was observed in the infected region using IFA (Figure 1A). Simultaneously, viral genome DNA was extracted from the supernatant from cultures exhibiting a CPE, and the PRV gE gene was amplified via PCR using the primers gE-F/gE-R (Table S1). As shown in Figure 1B, the supernatant from cultures exhibiting a CPE was positive for the gE gene. Then, the viruses were purified via plaque purification, with homogeneity monitored using the plaque sizes, and the isolates were named SX1910 and SX1911. We also tested the multistep growth and plaque size of the two isolates, and found that the growth kinetics of SX1910 was generally similar to SX1911, and the plaque sizes of SX1910 were significantly larger than those of SX1911.

To test the pathogenicity of the two isolates, mice were infected with SX1910 or SX1911 to determine the LD_50_. As shown in Table 2, at the same dose, mice inoculated with SX1910 died much earlier than mice inoculated with SX1911. The LD_50_ of each strain was calculated as 10^3.84^ TCID_50_ (SX1910) and 10^4.42^ TCID_50_ (SX1911), respectively, indicating that the pathogenicity of SX1910 was higher than that of SX1911.

### 3.2. Genomic Sequencing and Phylogenetic Analysis

The complete genome sequences of SX1910 and SX1911 were determined using the high-throughput Illumina platform. Following assembly and annotation, there were 69 open reading frames (ORFs) in both isolates. The complete genomes of the SX1910 and SX1911 strains encompass 143,186 bp and 143,263 bp with GC contents of 73.71% and 73.75%, respectively. To search for the aa variations between SX1910 and SX1911, all protein-coding regions of the SX1911 strain were compared with SX1910. Sequence comparison revealed that the 19 viral proteins displayed variations in the SX1911 strain (Table 3), and most of these proteins are associated with viral DNA replication or virulence.

**Figure 2:**
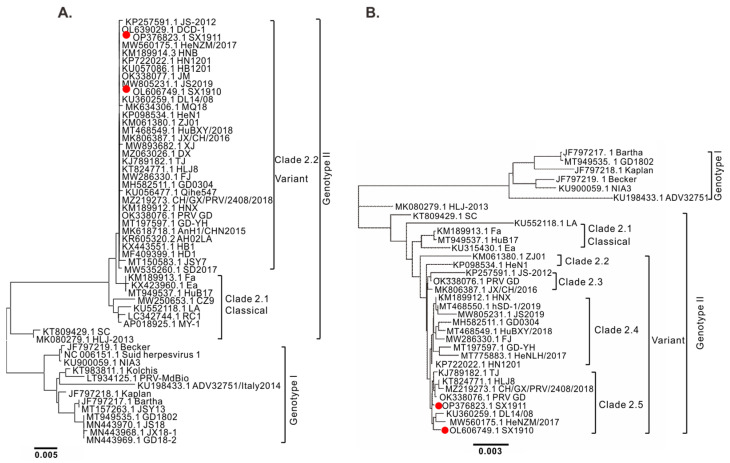
Phylogenetic analysis of PRV based on gC sequences and whole-genome sequences. (**A**) Phylogenetic analysis of PRV based on gC gene sequences using MEGA 5.1 software with the neighbor-joining algorithm, 1000 bootstrap replicates and the Kimura 2-parameter substitution model. (**B**) Phylogenetic analysis of PRV based on whole-genome sequences using Geneious Prime software (version 2022.2) with the neighbor-joining method, 100 bootstrap replicates and the Tamura–Nei genetic distance model.

To elucidate the genetic relationship of the two isolates, phylogenetic trees based on the gC sequences and the whole genomic sequences were constructed using a neighbor-joining method via MEGA 5.1 and Geneious Prime (version 2022.2) software. As shown in Figure 2A, the phylogenetic tree of gC showed that the PRV strains were divided into two genotypes. Genotype I is mainly composed of European–American strains, while Chinese strains belong to genotype II, which is consistent with previous studies [22]. Within genotype II, the PRV variants displayed the highest homology and were divided into the clade 2.2 group. Correspondingly, a similar result was found using whole-genome analysis (Figure 2B), but the variant strains were contained in four clades: clades 2.2, 2.3, 2.4 and 2.5. SX1910 and SX1911 were clustered into clade 2.5, together with strains HeNZM/2017, PRV GD, TJ and HLJ8. These results suggest that PRV variants are undergoing genetic variations but the genotype is unchanged.

### 3.3. The Extensive Variations That the Two Isolates Exhibit in the Protein-Coding Sequences UL5, UL36, US1 and IE180

**Figure 3:**
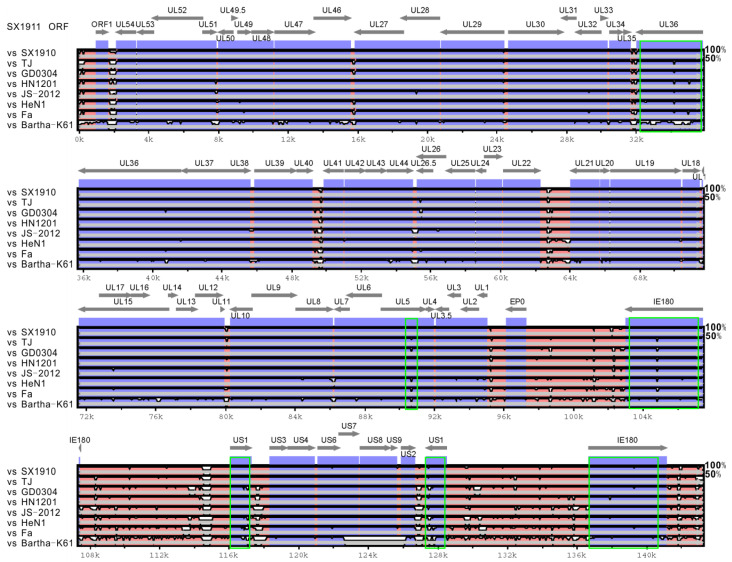
Comparison of the whole-genome sequence conservation between strain SX1911 and other PRV strains. Conservation scores were calculated from multiple sequence alignment, and the conservation score between any two genomes was plotted based on a sliding 100 bp window.

To explore the genetic variations in the two isolates, using the mVista genomic analysis tool with global LAGAN alignment, the SX1910 and SX1911 whole genomes were aligned with those of the reference strains from genotype I (Bartha-K61) and different clades of genotype II, including Fa (clade 2.1), HeN1 (clade 2.2), JS-2012 (clade 2.3), HN1201 (clade 2.4), GD0304 (clade 2.4) and TJ (clade 2.5). As shown in Figure 3 and Figure S1, compared with Bartha-K61, the variants carried gene deletions, insertions and substitutions scattered along the genome, which was consistent with previous studies [22]. Of note, among the variant strains, most genes were conserved, while the SX1910 and SX1911 strains showed extensive divergence in noncoding regions (internal/terminal repeat regions and intergenic sequences) and the protein-coding sequences UL5, UL36, US1 and IE180, which are associated with viral egress, DNA replication and transcriptional regulation.

**Figure 4:**
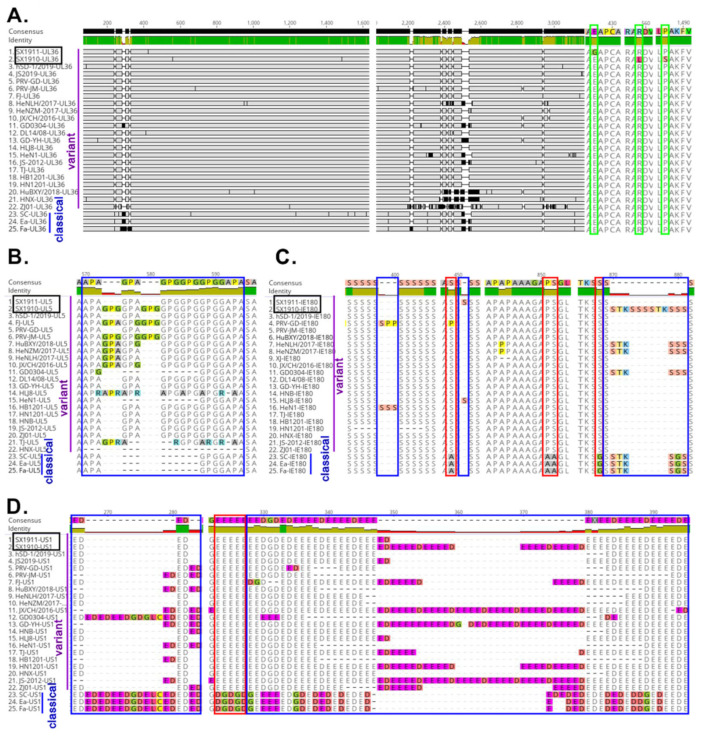
Multiple sequence alignment of divergent protein sequences among the genotype II strains. (**A**) UL36, (**B**) UL5, (**C**) IE180 and (**D**) US1. The novel aa mutations of the two isolates are indicated by a green box. The hypervariable aa regions are marked with blue boxes. The changes in conserved aa regions are marked with red boxes.

To further understand the characteristics of hypervariable genes, respectively, the viral UL36, UL5, US1 and IE180 protein sequences from 3 classical strains and 22 variant strains were aligned using Geneious Prime software with the Clustal Omega program. As shown in Figure 4A–D, compared with those of the reference strains, some novel aa mutations of the two isolates were observed. Specifically, two aa mutations (R559L and aa P1487S) in UL36 were identified in SX1910, and one aa mutation (E427G) was identified in SX1911. For IE180, a continuous 6 aa (SSSSTK) insertion at position 873–878 was observed in SX1910. SX1911 had an insertion of one serine (S) at position 451, identical to HLJ8. Notably, the indicated proteins had one or more hypervariable aa regions, such as UL36 (240–340 aa and 2200–2600 aa), UL5 (570–594 aa), IE180 (870–881 aa) and US1 (265–284 aa and 322–395 aa). Moreover, compared with those of classical strains, some conservative aa changes were also observed in variant strains. On the other hand, we also found that UL36 had many strain-specific aa mutations, and variation regions of the US1 protein (ICP22) mainly exhibited a continuous repeated aa deletion/insertion (ED or E) or substitution (E-G, D-E, G-E, or G-D). Overall, these results indicate that noncoding regions and the protein-coding sequences UL36, UL5, US1 and IE180 exhibited extensive variations, and these protein-coding sequences had one or more hypervariable aa regions and strain-specific aa mutations.

### 3.4. The Glycoproteins gB and gD of the Two Isolates Have New Amino Acid Mutations

**Figure 5:**
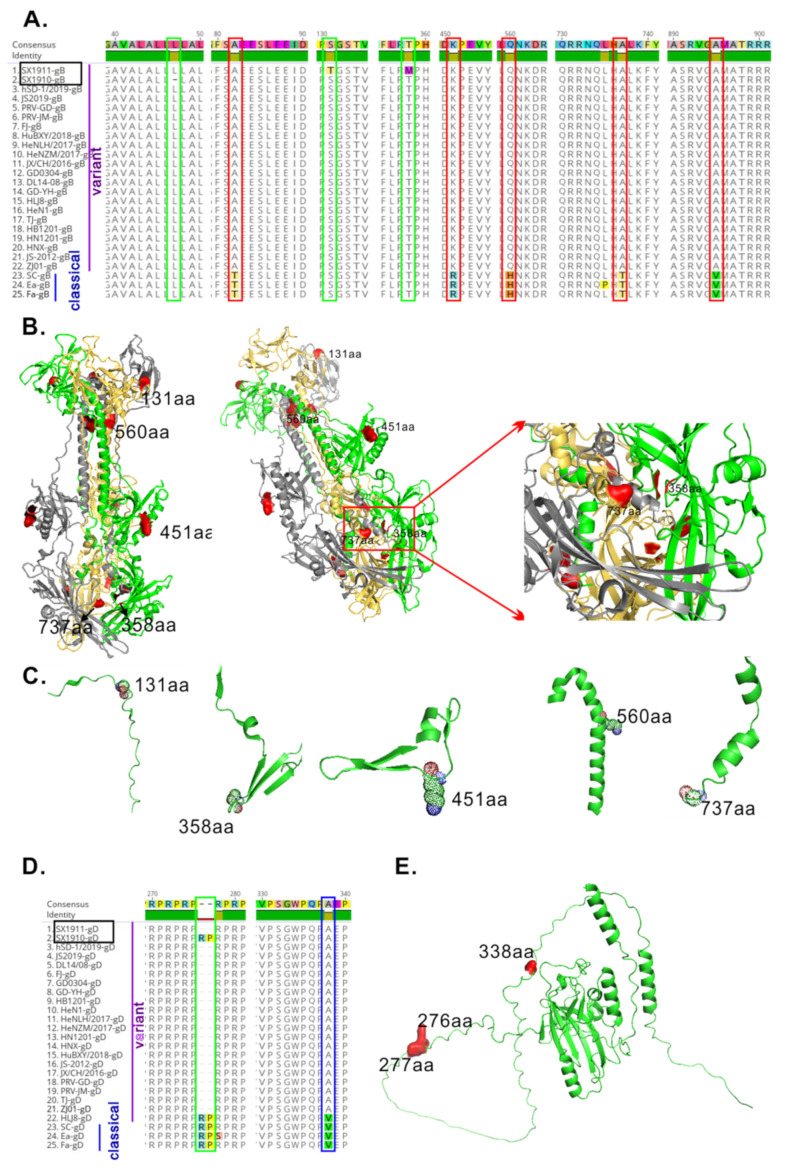
Analysis of the effect of aa mutations on gB and gD antigenicity, determined via bioinformatics. (**A**) Multiple sequence alignment of gB sequences among the genotype II strains. The novel aa mutations of the two isolates are indicated by a green box. The hypervariable aa regions are marked with blue boxes. The changes in conserved aa regions are marked with red boxes. (**B**) The position of aa mutations of surface residues in the structure of the PRV gB trimer (PRV strain HN1201, PDB ID: 5ys6) is shown with a cartoon representation. (**C**) Review of aa mutations in the gB the secondary structure. (**D**) Multiple sequence alignment of gD sequences among the genotype II strains. The novel aa mutations of the two isolates are indicated by a green box. The hypervariable aa regions are marked with blue boxes. The changes in conserved aa region changes are marked with red boxes. (**E**) The position of aa mutations of surface residues in the structure of the PRV gD is shown with a cartoon representation. Additionally, the gD structure was predicted via AlphaFold 2 combined with PRV HN1201 the gD structure (7–250 aa, PDB ID: 5X5V).

PRV glycoproteins play an important role in promoting virus entry, modulating virulence and inducing the immune response. To this end, we further explored the variations in gB, gC and gD of the isolated strains. In contrast with those of other variant strains, the major novel mutations occurred in gB and gD (Figure 5A,E), while gC was highly conserved. In gB alignment, two aa substitutions (S131T and T358M) in SX1911 and one aa (L) deletion at position aa 47 in SX1910 were identified. Moreover, a 2 aa (RP) insertion at position 276–277 was observed in gD of SX1910, identical to the classical strains and variant strain HLJ8. To determine whether the variation in gB and gD affects immunogenicity, we also examined the structure of the gB and gD proteins using PyMOL software (Schrodinger, Inc., New York, NY, USA). After removing the signal peptide (1–58 aa) and intracellular region (820–915 aa) of gB, we found that the mutant aa of SX1911 at position 131, which is located in coil structures, was on the surface of the molecule. Additionally, compared with those of classical strains, the two conserved mutant residues (aa 451 and 737) of the variant strains exhibited a similar result (Figure 5B,C). Furthermore, the RP at position 276–277 of gD in SX1910 was also located on the surface of the molecule (Figure 5E). These results suggest that the variation in the glycoproteins gB and gD might be involved in immunogenicity or antigenicity changes.

### 3.5. Construction and Biological Characterization of gE/gI-Deleted Virus of SX1911

**Figure 6:**
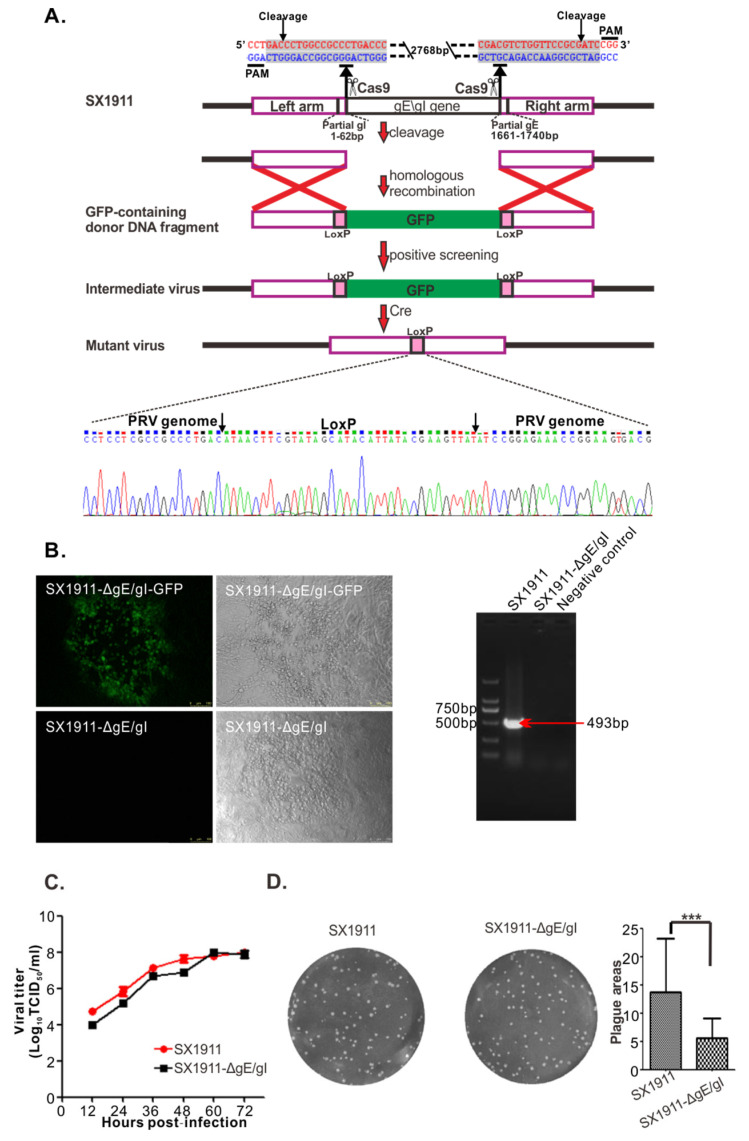
Construction and identification of the mutant virus SX1911-ΔgE/gI. (**A**) Strategy for constructing SX1911-ΔgE/gI using the CRISPR/Cas9 and LoxP systems. Two sgRNAs were designed to guide Cas9 to delete the gE and gI genes, and GFP was used for both positive and negative screening of mutant virus production. (**B**) Identification of SX1911-ΔgE/gI via IFA and PCR targeting the gE gene. (**C**) Multistep growth curve of SX1911 and SX1911-ΔgE/gI in Vero cells. (**D**) Plaque sizes of SX1911 and SX1911-ΔgE/gI in Vero cells. Data are presented as the mean ± SD, and an asterisk indicates a significant difference between SX1911 and SX1911-ΔgE/gI. ***: *p* < 0.001.

In our study, a mutant SX1911 virus lacking the gE and gI genes was generated using the CRISPR/Cas9 and Cre-loxp systems and named SX1911-ΔgE/gI (Figure 6A,B). Then, the biological characteristics of SX1911-ΔgE/gI were tested in Vero cells. Analysis of the multistep growth kinetics of SX1911-ΔgE/gI showed a replication efficiency similar to that of SX1911 (Figure 6C). The sizes of plaques formed by SX1911-ΔgE/gI were considerably reduced compared to those of its parental virus SX1911 (Figure 6D). When the pathogenicity of the mutant virus was tested in mice, SX1911-ΔgE/gI exhibited lower virulence than SX1911. According to our analysis using the Reed–Muench method, the LD_50_ values of SX1911-ΔgE/gI were 38-fold those of SX1911 (Table 1).

### 3.6. Bartha-K61 Provides a Comparable Protection Range to SX1911-ΔgE/gI

**Figure 7:**
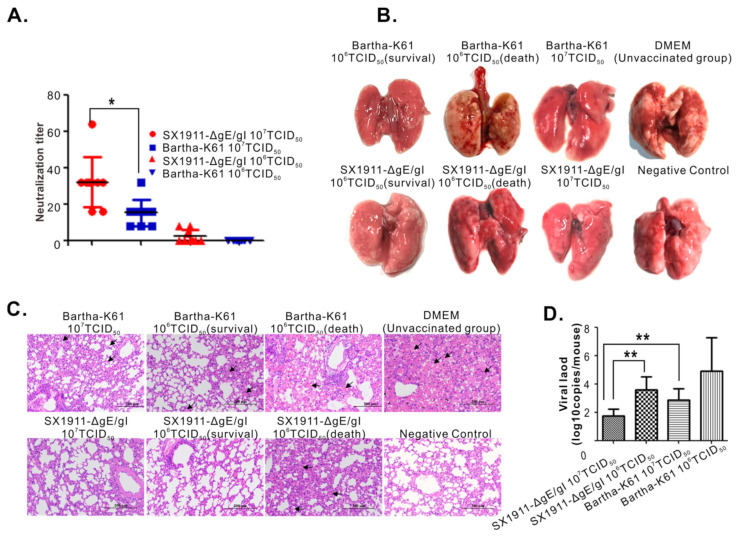
Protection efficiency analysis of SX1911-ΔgE/gI and Bartha-K61. (**A**) Neutralizing antibody titers against SX1911. Mouse sera were collected at 28 days post-immunization (dpi), and neutralizing antibodies were quantified in 96-well plates. (**B**) Gross lesion changes in immunized mice following challenge with SX1911. Lungs were collected and subjected to pathological examination at 14 days post-challenge (dpc). (**C**) Histopathological lesions of immunized mice following challenge with SX1911. The lungs were fixed, sectioned and stained with hematoxylin and eosin (HE) (200× magnification). (**D**) The tissue viral load of PRV in the lungs determined via qPCR. Data are presented as the mean ± SD, and an asterisk indicates a significant difference. *: *p* < 0.05; **: *p* < 0.01.

It has been reported that the Bartha-K61 vaccine cannot provide effective protection against PRV variants [18,19,20,21]. To investigate the protection efficiency of SX1911-ΔgE/gI, 6-week-old Kunming mice were vaccinated with 200 μL of either inactivated SX1911-ΔgE/gI or inactivated Bartha-K61 at doses of 10^6^ TCID_50_ or 10^7^ TCID_50_ via both the subcutaneous and intramuscular routes. After immunization, all mice displayed a good mental state, with normal appetite and no clinical symptoms, suggesting that these vaccines had no side effects on the mice. Serum samples were collected at 28 dpi, and neutralizing antibody levels were measured via a neutralization test. As shown in Figure 7A, the vaccines at a dose of 10^7^ TCID_50_ could effectively induce the mice to produce neutralizing antibodies, while the neutralizing antibody titers of the SX1911-ΔgE/gI group were significantly higher than those of Bartha-K61.

At 28 dpi, except for the negative controls, all mice were inoculated with SX1911 via the subcutaneous route at a highly lethal dose of 10^6^ TCID_50_. Following the challenge, the DMEM group (unvaccinated group) began to exhibit obvious symptoms 72 h later, such as apathetic mood, rough hair disorder, loss of appetite, weight loss, and constant biting of the injection sites. For the immunized groups, mice immunized with a dose of 10^7^ TCID_50_ did not show obvious clinical symptoms, except moderate or slight depression in the Bartha-K61 group. Three mice died and displayed severe symptoms in the groups receiving a challenge dose of 10^6^ TCID_50_, including those immunized with SX1911-ΔgE/gI and Bartha-K61 (Table 4). At 14 dpc, all surviving mice were euthanized and necropsied, and the lungs were collected for pathological examination and viral load analyses. As shown in Figure 7B, postmortem necropsy showed that the dead mice receiving an immunization dose of 10^6^ TCID_50_ had obvious consolidation and severe hemorrhage in the lung. Mice in the SX1911-ΔgE/gI group did not show a significant difference from those in the Bartha-K61 group. For the mice immunized with a dose of 10^7^ TCID_50_, moderate or mild hemorrhagic lesions were observed in the Bartha-K61 group. However, we did not observe visible pathological lesions in the SX1911-ΔgE/gI group. Next, we also examined the histopathological changes via HE staining (Figure 7C), and found that the mice in the Bartha-K61 group developed more severe microscopic lesions than the mice in the SX1911-ΔgE/gI group, such as alveolar septal capillary dilatation, hemorrhage, congestion and alveolar destruction. Thus, the pathological examination revealed that SX1911-ΔgE/gI could more effectively reduce organ lesions compared with Bartha-K61. On the other hand, we assessed the viral load in the lungs using quantitative real-time PCR with primers targeting the gene encoding gB (Figure 7D). The results showed that a high immunization dose (10^7^ TCID_50_) significantly reduced the viral tissue load in the lungs compared with that after low-dose (10^6^ TCID_50_) immunization. Interestingly, at an immunization dose of 10^7^ TCID_50_, the viral load in the lungs (PRV genome copies) of mice in the SX1911-ΔgE/gI group was much lower than that the lungs of mice in the Bartha-K61 group. Overall, these data indicated that SX1911-ΔgE/gI-vaccinated mice were protected within a comparable range to Bartha-K61-vaccinated mice. Additionally, a higher dose of inactivated Bartha-K61 protected the mice from lethal SX1911 challenge, while a lower neutralization titer, higher viral load and more severe microscopic lesions were displayed in Bartha-K61-vaccinated mice.

## 4. Discussion

Since 2011, highly virulent PRV variants have caused substantial economic losses to the swine industry in China [35]. In recent years, an increasing number of reports have indicated that PRV variants are undergoing extensive aa mutations and intragenotype and intergenotype gene recombination. In this report, to surveil the genetic variation in PRV field strains, we analyzed the genetic variation in two novel PRV variant strains (SX1910 and SX1911) isolated in Shanxi Province, China. We revealed the following findings. (i) The two isolates exhibited the greatest foci of divergence in noncoding regions and the protein-coding regions UL5, UL36, US1 and IE180, which are associated with viral egress, DNA replication and transcriptional regulation. (ii) The glycoproteins gB and gD of the isolated strains had novel aa mutations, and these mutations were mainly located on the surface of the molecule, corresponding to a higher antigenic index. (iii) High doses of Bartha-K61 alone could provide relatively better immunity in mice, and its protection efficiency exhibited a comparable range to SX1911-ΔgE/gI. The relevant significance and insights of this study are discussed below.

Phylogenetic characterization revealed that PRV variants were classified into genotype II, exhibiting remarkable divergence from genotype I strains, including Bartha-K61 and Becker [22,23,36,37,38]. Similarly, our study also revealed that PRV SX1910 and SX1911 were variant strains. However, SX1910 and SX1911 were also undergoing genetic variation and were clustered into clade 2.5. In addition, Hu et al. found that a unique genetic clade of PRV variants was detected via phylogenetic analysis, implying that PRV variants continuously evolved between 2012 and 2017 in China [27]. Of note, the HLJ-2013 strain is a recombinant virus that emerged from a recombination event between genotype I and genotype II strains, so HLJ-2013 is located in an independent branch [39]. Similar results were found in another study based on phylogenetic analysis of the whole genome [37].

Extensive genetic variations in the variant strains are scattered along the genome, in comparison to Bartha-K61 or Becker from genotype I [22]. However, according to the genotype II strain genome alignment, the hypervariable regions of the SX1910 and SX1911 genomes predominantly occurred in internal and terminal repeat regions, intergenic sequences and some protein-coding sequences, such as UL36, UL5, US1 and IE180. Notably, the viral proteins UL36, UL5, US1 and IE180 are responsible for viral egress, DNA replication and gene transcriptional regulation [2,40,41,42,43]. Each protein contains one or more hypervariable aa regions displaying aa deletions, insertions and substitutions. Previously, a study indicated that aa 2087–2796 in UL36 (VP1/2) is required for PRV Becker strain virulence and retrograde axon transport in vivo [42]. Interestingly, our study showed that the hypervariable aa 2200–2600 region of UL36 (VP1/2) was located within the functional aa 2087–2796 region. This finding suggests that the variations in aa 2200–2600 might be associated with virulence attenuation or enhancement in PRV variants. US1 (ICP22) is involved in transactivation and regulatory functions in related alphaherpesviruses [40]. Ye et al. found that PRV variants exhibited the highest variation rate among all viral proteins [22]. Moreover, our study also showed that US1 protein (ICP22) variations mainly exhibited a continuous repeated aa deletion/insertion (ED or E) or substitution (E-G, D-E, G-E, or G-D). In addition, in herpes simplex virus type I (HSV-1) and duck plague virus (DPV), ICP22 is required for efficient viral replication and pathogenicity [44,45,46,47]. Therefore, it would also be interesting to explore the role and significance of US1-coding sequence variability in the future. Of note, the “hypervariable regions” also might be due to different types of repeated sequence which are not only instable but also problematic when using NGS techniques. Therefore, some of the variations in the genomes are probably due to sequencing (assembly) errors. The glycoproteins gB, gC and gD are the key proteins involved in virus entry and the induction of neutralizing antibody production or protective immunity [2]. Variations in gB, gC and gD contribute to PRV variant escape from the immunity provided by Bartha-K61 [21,48,49]. To this end, we also detected variations in gB, gC and gD in the two isolates. The results showed that most novel aa mutations of gB and gD are mainly scattered on the surface of the protein molecule. Thus, we speculated that the aa mutations of gB or gD in the two isolates might cause antigenic drift.

It has been reported that PRV variants are highly virulent [15,16,17]. In a mouse model, the LD_50_ values of variant strains JS-2012 and TJ were 10^3.0^ TCID_50_ and 10^2.3^ TCID_50_ [15,48], respectively. However, in our study, we found that the LD_50_ values of SX1910 (10^3.84^ TCID_50_) and SX1911 (10^4.42^ TCID_50_) in mice were lower than those of the JS-2012 and TJ strains, indicating that the two isolates might be less pathogenic PRV variants. Additionally, Zhou et al. isolated a moderately pathogenic PRV variant strain, SD1401, in Shandong Province, China [50]. These data suggested that some PRV variants might be undergoing low-pathogenicity evolution, which might lead to genetic diversity in variant strains in pig farms.

Previous studies indicated that the Bartha-K61 vaccine could not provide effective protection against PRV variants [18,19,20,21]. In contrast, we provided evidence that Bartha-K61 could provide better protection via a high immunization dose. In addition, Wang et al. found that Bartha-K61, at a dose of 1 × 10^5^ TCID_50_ per animal, protected pigs from sublethal challenge with the variant strain XJ5, whereas lower doses of Bartha-K61 alone did not protect pigs against this challenge [51]. The clinical protective efficiency usually depends on the immune responses induced by the vaccine and the virulence of the virus. Thus, in addition to the immune response induced by the high dose of vaccine, we also speculated that the variant strain SX1911 is a moderately pathogenic strain, so SX1911 might not overwhelm the host immunity provided by Bartha-K61. To date, based on the genetic backbone of variant strains, more novel PRV vaccines have been constructed or licensed and put on the market [52,53,54]. These vaccines showed better protection than Bartha-K61 against variant strains from the field. Surprisingly, our studies showed that immunization with SX1911-ΔgE/gI and Bartha-K61 resulted in equal mortality when the mice were challenged with SX1911. However, it should be noted that a lower neutralization titer, higher viral load and more severe microscopic lesions were displayed in Bartha-K61-vaccinated mice. Collectively, our results provide useful information for vaccination interventions when choosing the Bartha-K61 vaccine to eradicate PRV variants. Moreover, SX1911-ΔgE/gI is a promising vaccine candidate for the effective control of the current epidemic of PR in China.

## Figures and Tables

**Table 1:** Clinical scores in the mice after PRV infection.

Symptoms	Clinical Scores ^a^
Normal	0
Ruffled hair	1
Depression	1
Anorexia	1
Moderately labored breathing	1
Urgent breathing	2
Itching	2
Skin biting	3
Paralysis	3
Death	3
Total scores	17

**^a^** Clinical signs are represented by +++: serious (11 ≤ total average score ≤ 17), ++: moderate (5 ≤ total average score ≤ 10), +: mild (0 ≤ total average score ≤ 4) and /: none.

**Table 2:** Statistical analysis of mice infected with PRV.

Groups	Amounts	Doses (TCID_50_/mL)	Clinical Signs ^a^	Mortality (Mean Days to Death)	LD_50_
SX1910	10	10^6^	+++	10/10 (2.98)	10^3.84^
	10	10^5^	+++	8/10 (3.51)	
	10	10^4^	++	6/10 (4.08)	
	10	10^3^	+	2/10 (4.25)	
SX1911	10	10^6^	+++	10/10 (3.25)	10^4.42^
	10	10^5^	+++	7/10 (3.99)	
	10	10^4^	++	3/10 (4.16)	
	10	10^3^	+	1/10 (4.25)	
SX1911-ΔgE/gI	10	10^6^	++	5/10 (4.07)	10^6.00^
	10	10^5^	+	2/10 (6.68)	
	10	10^4^	/	0/10	
	10	10^3^	/	0/10	
DMEM	10	0.1 mL	/	0/10	

**^a^** Clinical signs are represented by +++: serious, ++: moderate, +: mild and /: none.

**Table 3:** Protein-coding variations of PRV SX1911, in comparison to PRV SX1910.

Protein Name ^a^	Amino Acid Residues Found in PRV SX1911, which Differ from PRV SX1910 ^b^
gK (UL53)	F124L
gN (UL49.5)	W42R
gB (UL27)	45 (+L), S131T, T358M
ICP18.5 (UL28)	G255A, 256 (+G)
ICP8 (UL29)	M178T
VP1/2 (UL36)	E404G, L536R, S1464P
UL37	D844G
RR1 (UL39)	G589V
UL43	G206D
Scaffold (UL26.5)	V269P
VP24 (UL26)	V515M
OBP (UL9)	L565, W723R
UL8	V5A, V293A, A532E
UL5	74–79 (PGGPAG > Δ)
ICP0 (EP0)	V345A
ICP4 (IE180)	Q98Δ, 869–880 (STKSSSSTKSSS > Δ), 448 (+S)
ICP22 (US1)	352–371(EEEEDEEEEDEEEEDEEED > Δ)
gD (US6)	280–281 (RP > Δ)
gE (US8)	V386A, G510S

^a^ Proteins are listed in order of occurrence along the genome. ^b^ single AA residue changes are written in standard format, including the SX1910 strain AA, its position and the AA residue found in the PRV SX1911 protein, e.g., S100P. Insertions are indicated by the AA position in SX1910, followed by “+” and the new AAs, e.g., 100 (+RR). Deletions are indicated by the symbol Δ. Sequential changes are combined and shown with the SX1910 strain AA positions are shown first, followed by the relevant SX1910 AA residues, and then “>”, and finally, the new alternative AA residues, e.g., 100–102 (RAR > EDA).

**Table 4:** Statistical analysis of the protection efficiency of SX1911-ΔgE/gI in mice.

Groups	Amounts	Immunization Doses (TCID_50_/mL)	Clinical Signs ^a^	Mortality (Mean Days to Death)
SX1911-ΔgE/gI	9	10^6^	+	3/9 (4.00)
SX1911-ΔgE/gI	9	10^7^	/	0/9
Bartha-K61	9	10^6^	+	3/9 (3.66)
Bartha-K61	9	10^7^	+	0/9
DMEM	9	0.2 mL DMEM	+++	9/9 (4.44)
Negative control	9	0.2 mL DMEM	/	0/9

**^a^** Clinical signs are represented by +++: serious, +: mild and /: none.

## Data Availability

All data generated or analyzed during this study are included in the published article.

## Supplementary Materials

The following supporting information can be downloaded at: https://www.mdpi.com/article/10.3390/v15061237/s1, Figure S1: Comparison of the whole-genome sequence conservation between strain SX1910 and other PRV strains; Table S1: Primers for amplification of genes in this study; Table S2: The PRV reference strains used in this study; Table S3: oligos to construct the sgRNA plasmid used in this study.
