# Supplementary material for: Genomic Characterization and gE/gI-Deleted Strain Construction of Novel PRV Variants Isolated in Central China
Source: Viruses. 2023 May 25;15(6):1237. doi: 10.3390/v15061237 (PMC10305206; doi:10.3390/v15061237)
Supplement: Supplementary file 1 [file viruses-15-01237-s001.zip › viruses-2381951-supplementary.pdf]

Supplementary Material

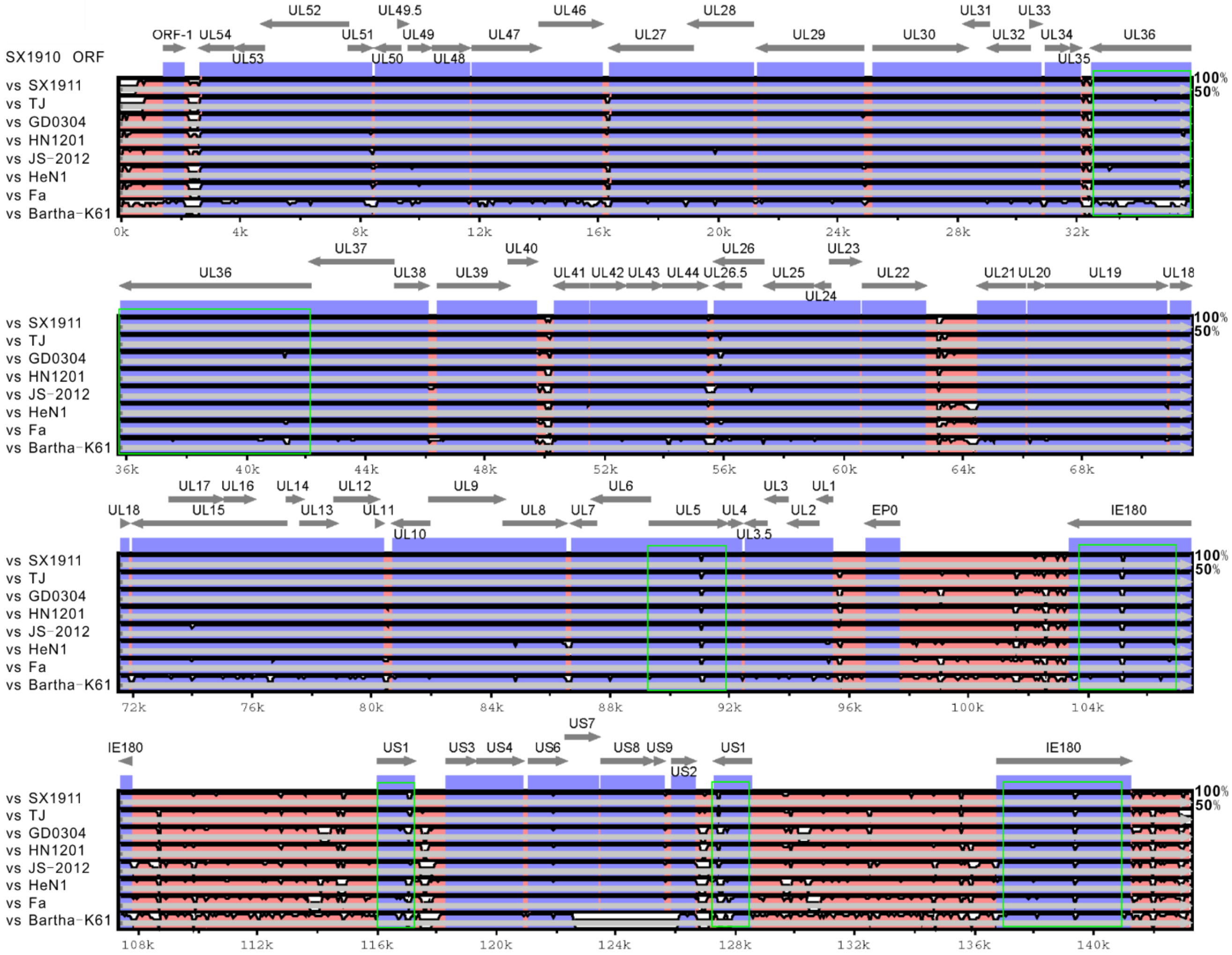

Supplementary Figure S1. Comparison of the whole-genome sequence conservation between strain SX1910 and other PRV strains. Conservation scores were calculated from a multiple sequence alignment, and the conservation score between any two genomes was plotted based on a sliding 100 bp window.

Table S1 Primers for amplification of genes in this study.

Primers	Sequences (5’-3’)	Gene name
gE-F	CCGCGGGCCGTGTTCTTTGT	Partial gE gene
gE-R	CGTGGCCGTTGTGGGTCAT	
ΔgE/gI-GFP-F	CCCCGCGGGGCTCCTCCTCGCCGCCCTGACATAACTTCGTATAGCATACATTATACGAAGTTATTAGTTATTAATAGTAATCAATTACGGGGTC	ΔgE/gI -GFP
ΔgE/gI-GFP-R	GTCCATTCGTCACTTCCGGTTTCTCCGGATATAACTTCGTATAATGTATGCTATACGAAGTTATAGATACATTGATGAGTTTGGACAAACCACA	
ΔgE/gI -left-F	CCGCACCGGGAGGTGGTGAACTACTGGTAC	ΔgE/gI-left arm
ΔgE/gI -left-R	GTCAGGGCGGCGAGGAGGAGCCCCGCGGGGAG	
ΔgE/gI -right-F	ATCCGGAGAAACCGGAAGTGACGAATGGAC	ΔgE/gI-right arm
ΔgE/gI -right-R	CTACACGTGCCTGGCGACGATGCCCCCGAG	

Table S2 The PRV reference strains used in this study.

PRV strains	Country	Year	GenBank accession No.
Bartha-K61	Hungary	1961	JF797217.1
Kaplan	Hungary	/	JF797218.1
NIA3	United Kingdom	2008	KU900059.1
Becker	USA	/	JF797219.1
DUL34Pass	Germany	/	JQ809330.1
ADV32751/Italy2014	Italy	2014	KU198433.1
Kolchis	Greece	2010	KT983811.1
PRV-MdBio	Hungary	2017	LT934125.1
Suid herpesvirus 1	USA	2003	NC_006151.1
TPA_exp	USA	2003	BK001744.1
SC	China	1986	KT809429.1
Ea	China	1993	KU315430.1
LA	China	1997	KU552118.1
Fa	China	1962	KM189913.1
HNB	China	2012	KM189914.3
TJ	China	2012	KJ789182.1
HNX	China	2012	KM189912.1
HeN1	China	2012	KP098534.1

ZJ01	China	2012	KM061380.1
HLJ8	China	2013	KT824771.1
GD-YH	China	2014	MT197597.1
GD0304	China	2015	MH582511.1
HeNLH/2017	China	2017	MT775883.1
PRV-JM	China	2017	OK338077.1
PRV-GD	China	2021	OK338076.1
CH/GX/PRV/2408/2018	China	2018	MZ219273.1
JS2019	China	2019	MW805231.1
HuBXY/2018	China	2018	MT468549.1
HeNZM/2017	China	2017	MW560175.1
FJ	China	2019	MW286330.1
HuB17	China	2020	MT949537.1
GD1802	China	2020	MT949535.1
JX/CH/2016	China	2016	MK806387.1
HLJ-2013	China	2013	MK080279.1
DL14/08	China	2014	KU360259.1
HN1201	China	2012	KP722022.1
JS-2012	China	2012	KP257591.1
HB1201	China	2012	KU057086.1

DCD-1	China	2017	OL639029.1
DX	China	2012	MZ063026.1
AnH1/CHN2015	China	2015	MK618718.1
AH02LA	China	2011	KR605320.2
JSY7	China	2018	MT150583.1
JSY13	China	2018	MT157263.1
SD2017	China	2017	MW535260.1
MY-1	Japan	2015	AP018925.1
RC1	Japan	2016	LC342744.1
CZ9	China	2019	MW250653.1
HB1	China	2012	KX443551.1
HD1	China	2017	MF409399.1
JX18-1	China	2018	MN443968.1
GD18-2	China	2018	MN443969.1
JS18	China	2018	MN443970.1
XJ	China	2015	MW893682.1
MQ18	China	2018	MK634306.1
Qihe547	China	2014	KU056477.1
hSD-1/2019	China	2019	MT468550.1

Table S3 oligos to construct the sgRNA plasmid used in this study.

sgRNA oligos	Sequences (5'-3')	sgRNA plasmids
gE-sgRNA-F	CACCCGACGTCTGGTTCCGCGATC	pX335-gE
gE-sgRNA-R	AAACGATCGCGGAACCAGACGTCG	
gI-sgRNA-F	CACCGGGTCAGGGCGGCCAGGGTC	pX335-gI
gI-sgRNA-R	AAACGACCCTGGCCGCCCTGACCC	
